# Development of a non‐lethal hydrogen peroxide treatment for surveillance of *Gyrodactylus salaris* on trout farms and its application to testing wild salmon populations

**DOI:** 10.1111/tbed.13263

**Published:** 2019-06-18

**Authors:** Mark A. Thrush, Tom Hill, Nick G. H. Taylor

**Affiliations:** ^1^ Centre for Environment, Fisheries and Aquaculture Science (Cefas) Weymouth UK

**Keywords:** aquatic, biosecurity, disease, fish pathogens, *Gyrodactylus salaris*, surveillance

## Abstract

This study documents the development of a non‐lethal sampling method to recover gyrodactylid parasites from large numbers of fish that will underpin an improved surveillance strategy for *Gyrodactylus salaris*. A review of published literature identified over 80 compounds that have previously been tested against gyrodactylids or closely related parasite species. Five safe and relatively fast‐acting compounds were selected for testing to determine their efficiency in removing gyrodactylids from host fish in small‐scale aquaria trials using three‐spined stickleback infected with *Gyrodactylus gasterostei* as a model host–parasite system. The most effective compound was hydrogen peroxide; short‐duration exposure (3 min) achieved a parasite detection sensitivity of 80%–89%. The practicality of exposing farmed salmonids to hydrogen peroxide for *G. salaris* surveillance was tested in the field by conducting a parasite recovery trial using a brown trout stock endemically infected with *G. truttae* and *G. derjavinoides* and comparing this to the whole‐body examination procedure currently conducted by UK authorities. Significantly more parasites were recovered after exposing fish to hydrogen peroxide and filtering the treatment solution than by direct whole‐body examination of killed fish (mean: 225 vs. 138 parasites per fish). The gyrodactylid recovery rate of the two methods was 84.6% and 51.9%, respectively. A comparison of timings for the two methods indicated scope for significant time savings in adopting the chemical screening method. The study demonstrated that hydrogen peroxide bath treatment may be successfully applied to the surveillance of gyrodactylid parasites and established as a non‐lethal method for sampling farmed and wild fish. This approach has the potential to reduce resources required to collect and isolate parasites for diagnostic testing and improve the sensitivity and confidence of surveillance programmes designed to demonstrate freedom from disease, thus underpinning a robust and defensible surveillance strategy for *G. salaris* for the UK aquatic animal disease contingency plan.

## 1 INTRODUCTION

*Gyrodactylus salaris* is a monogenean parasite of Atlantic salmon (*Salmo salar*) that can cause high levels of infection and mortality in pre‐smolts and may reduce the salmon population in a river to <5% within 5 years (Johnsen & Jensen, 1991). Its introduction into Norway in the early 1970s resulted in the collapse of wild salmon populations across the country (Bakke, Cable, & Harris, 2007), and to date, 50 rivers have been infected (Hytterød et al., 2018). Eradication programmes (using rotenone, or more recently acidified aluminium sulphate in combination with rotenone applied in inaccessible areas) have been conducted on 43 rivers, 32 of which have been confirmed as being successful. Currently, only the UK, Eire, the Faroe Islands and Iceland have recognized national *G. salaris*‐free status, and the potentially catastrophic impact of this parasite makes it arguably the most important disease threat to UK salmon populations (Peeler, Gardiner, & Thrush, 2004). Consequently, efforts have been made by UK Government to develop contingency plans to contain and limit the parasite's impact should it be introduced (Anon, 2018). In the event of an introduction, effective surveillance will be key to determining the geographic distribution of the parasite and containing it.

Different strains of *G. salaris* are known to exist, with a number of DNA taxonomy studies demonstrating extensive intraspecific differentiation (Hansen, Bachmann, & Bakke, 2003; Hansen, Bakke, & Bachmann, 2007; Meinilä, Kuusela, Ziętara, & Lumme, 2004). These have varying virulence and host preference characteristics, including examples with low virulence against Atlantic salmon (Lindenstrøm, Collins, Bresciani, Cunningham, & Buchman, 2003; Olstad, Robertsen, Bachmann, & Bakke, 2007). Importantly, strains of *G. salaris* with high virulence to Atlantic salmon can also infect, reproduce and permanently survive at low prevalence on rainbow trout (*Oncorhynchus mykiss*) without causing clinical disease (Bakke, Jansen, & Kennedy, 1991), and have been shown to reproduce and persist for at least 110 days, under experimental conditions, on brown trout (*Salmo trutta*) (Paladini et al., 2014); therefore, establishing the infection status of farmed trout stocks will be a critical element of any surveillance strategy for this parasite. Rainbow and brown trout are currently reared on 150 and 672 farm and fishery sites, respectively, in 115 river catchments in England and Wales (Cefas Starfish Database, 2018^1^), and the movement of live fish (e.g. for restocking and the supply of fingerlings for table production) will be the most likely route by which the parasite will spread through the UK if an introduction was to occur (Peeler et al., 2004). However, the low prevalence and abundance of *G. salaris* on these species means it is likely to go unnoticed in farmed populations and may silently spread for many months until significant outbreaks have occurred in wild salmon stocks. Modelling work conducted by Cefas demonstrated that, due to the complexity of the inter‐farm trout movement network, the likelihood of a severe outbreak of disease in England and Wales (involving 10 or more river catchments) would be very high should the parasite not be detected for more than 3 months (Thrush & Peeler, 2006). Further cross‐border connectivity in UK trout sectors (Jones et. al., 2019) suggests that an epidemic would be likely to extend into Scotland. This situation would result in high numbers of farm sites falling under suspicion of infection, all of which would require testing under current legislation (2006/88/EC; Anon, 2006). Furthermore, in populations where the prevalence of *G. salaris* is low, surveillance sensitivity will be reduced so that large sample sizes will be required to establish freedom from disease. A study conducted by Cefas demonstrated the difficulty in isolating non‐pathogenic gyrodactylid species in a farm survey (no gyrodactylids were recovered from 16 of 27 trout farms sampled) and highlighted the unacceptable time effort required to remove parasites from fish for identification (Anon, 2009). Current estimates of sample sizes required to demonstrate the absence of pathogenic parasites from a rainbow trout farm with an acceptable level of confidence (95%) stand at 295 fish (for whole‐body examination) or 595 fish (using fin samples; Peeler & Oidtmann, 2008), which clearly has very high ethical and resource implications should it be necessary to investigate more than a small number of sites (all fish would currently need to be killed for testing).

Clearly, a more efficient method of detecting pathogenic gyrodactylids is urgently required. There is increasing interest in the application of environmental DNA (eDNA) analysis in monitoring programmes (Bass, Stentiford, Littlewood, & Hartikainen, 2015). Environmental DNA is a term that may be used to define the wide range of genetic material present in environmental samples, from extracellular DNA to whole organisms (Barnes & Turner, 2016), and its analysis may be particularly relevant to the detection of parasites that are genetically diverse but morphologically difficult to distinguish. The potential for this approach has been established as a surveillance tool for *G. salaris* (Rusch et al., 2018). However, the unambiguous identification of parasites for notifiable disease management still requires the recovery of specimens. To address the efficiency of parasite collection specifically, this paper details the development of a non‐lethal bath treatment method to recover gyrodactylid parasites from large numbers of fish. A review of the available scientific literature was conducted to identify chemical compounds likely to rapidly remove gyrodactylids from their hosts whilst being safe to fish. The efficacy of the most promising candidate compounds was tested in small‐scale trials in static aquaria using three‐spined stickleback (*Gasterosteus aculeatus*) as a model host–parasite system to determine the proportion of parasites abandoning their hosts in response to short‐duration chemical exposure. In the UK, the stickleback plays host to a number of gyrodactylid species, including *Gyrodactylus gasterostei*, *Gyrodactylus alexanderi* and *Gyrodactylus arcuatus* (Harris, 2008; Raeymaekers, Huyse, Maelfait, Hellemans, & Volckaert, 2008), and its ubiquitous distribution makes sourcing infected fish relatively easy. Previous experience has also shown this to be a suitable experimental animal due to its ease of culture, small size and robustness (Katsiadaki et al., 2007).

The technique was then scaled up by assembling a robust and practical apparatus for exposing small groups of farmed salmonids to a chemical treatment that was tested in the field by conducting a parasite recovery trail. Brown trout were used in the farm trial, a host to *G. salaris* in the parasite's current geographical range (Paladini et al., 2014) and to other gyrodactylid species which are widespread in the UK (including *G. truttae* and *G. derjavinoides*). The study objectives were to: (a) refine and quantify a method for isolating and concentrating parasites removed from groups of fish by chemical treatment for identification; (b) establish the gyrodactylid recovery rate of the method when deployed on a commercial trout farm and compare this to that of the whole‐body examination method currently conducted by the Competent Authority^2^ and (c) review the time and resource requirements of the two techniques. The chemical exposure and parasite recovery steps of the methodology presented here represent the basis of a non‐lethal sampling procedure. However, to establish the gyrodactylid recovery rate of the technique, experimental fish were subsequently euthanized to determine the number of parasites that remained attached to their host after chemical treatment.

## 2 MATERIALS AND METHODS

### 2.1 Literature review and chemical selection

A review of chemical compounds previously tested against *Gyrodactylus* spp. or closely related parasite species was conducted. Scientific literature was systematically searched through Web of Knowledge and Google Scholar using combinations of the following search terms: Monogenea, Monogenean, Monopisthocotylea, Gyrodactylus, Dactylogyrus, Platyhelminthes, Benedenia, Polyopisthocotylea, Zeuxapta, Neobenedenia, Diclidophora, Protopolystoma, treatment, control, management, toxicity and chemical. A database was compiled summarizing the compounds tested, the dose and duration used, whether an in vivo study had been conducted, and if so, which host species it was tested on. Also recorded was the parasite species each compound was tested on, its effectiveness and whether any adverse effects on the host were observed. Five compounds were selected from the data set that could be taken forward for the assessment of their efficiency in removing gyrodactylids from host fish as part of a surveillance programme: formalin (formaldehyde), hydrogen peroxide (H_2_O_2_), Praziquantel, sodium chloride and sodium percarbonate. Selection was based on the following attributes: chemicals were effective against the target parasite species, were safe for host species and human users, and showed relatively acute efficacy.

### 2.2 Chemical efficacy study

#### 2.2.1 Study animals

Two groups of 150 sticklebacks were obtained from sites on the River Bourne (Kent) and River Allan (Stirlingshire). Gross presence of *Gyrodactylus* spp. was confirmed using a Leica M125 stereomicroscope. To ensure sufficient experimental animals for the study, the infected wild fish were co‐habited in a single tank with 200 naive, laboratory‐raised stickleback and held at 16 ± 1°C for 20 days to establish a suitable parasite culture for chemical testing. The origin of individual fish could subsequently be identified by size (mean lengths were approximately 30 and 60 mm for wild or laboratory‐reared fish, respectively). A representative sample of fish was regularly visually screened to monitor host welfare and parasite numbers to assess the progress of the co‐habitation challenge.

#### 2.2.2 Experimental challenge

The concentrations for sodium chloride and sodium percarbonate were informed by the chemical review. The concentrations for hydrogen peroxide and formalin were based on efficacy studies published by Buchmann and Kristensson (2003) and Rach, Gaikowski, and Ramsay (2011), respectively. Praziquantel is licensed for veterinary application and was sourced as a formulated product (Fluke‐Solve™ Aquarium) and was used as directed by the manufacturer's instructions.

Host animals were individually exposed to chemical bath treatments in two trials (Table 1). Live fish were netted from the rearing tank into a beaker containing approximately 200 ml of treatment solution. Following exposure, the host was removed from the treatment, euthanized (by schedule 1 method: concussion and destruction of the brain) and stored in 100% ethanol. The number of parasites in both the treatment solution and on the host was then counted separately (to determine the proportion of parasites removed from the host during the exposure period). Two control groups were included (live and euthanized fish exposed to de‐chlorinated water) to account for parasites detaching from dead hosts, mechanical removal from particularly active or stressed hosts, or during the euthanizing procedure.

**Table 1:** Exposure conditions and replicates for chemical efficacy testing in Trials 1 and 2

Treatment	Concentration	Exposure time (min)	Replicates	
			Trial 1	Trial 2
Formaldehyde (F)	20 ppm	3	10	–
Hydrogen peroxide (HP)	560 ppm	3	10	20
Praziquantel (P)	4 ppm	3	10	20
Sodium chloride (SC)	33 ppt	3	10	20
Sodium percarbonate (SP)	160 ppm	3	10	–
Control 1: dechlorinated water – Live fish (W)	n/a	3	10	20
Control 2: dechlorinated water – Dead fish (D)	n/a	3	10	–

#### 2.2.3 Parasite identification

Species identification was conducted using morphological and molecular techniques following the careful removal and isolation of parasites from their host's skin, as described by Shinn et al. (2010) (gyrodactylid parasites generally remain attached to their host when preserved in ethanol). For morphological assessment, individual parasites were placed on a glass microscope slide for examination by phased‐light microscopy and identified by characterization of the hamuli and marginal hooks located around the periphery of the opisthaptor (attachment organ). Nine parasites were also submitted for confirmatory molecular analysis. These were individually stored in G2 tissue digestion buffer (Qiagen) for subsequent identification by amplification and sequencing of the internal transcribed spacer (ITS) region (Matejusová, Gelnar, McBeath, Collins, & Cunningham, 2001).

### 2.3 Field validation

#### 2.3.1 A system for parasite recovery

**Figure 1:**
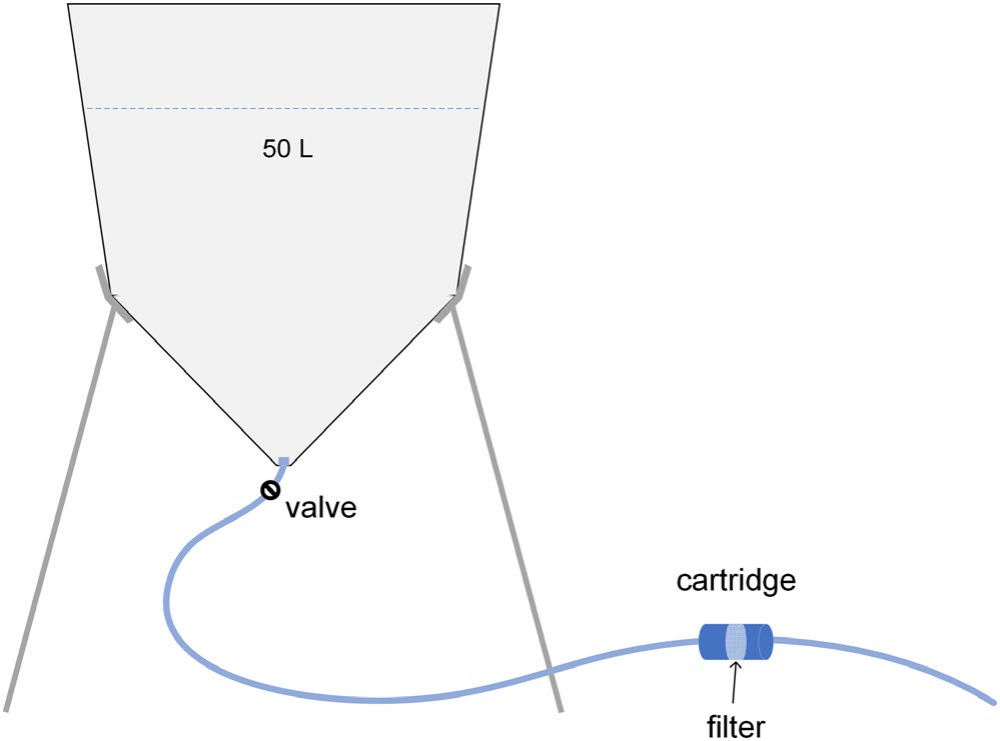
Apparatus used for exposing fish to hydrogen peroxide in the farm parasite recovery trial [Colour figure can be viewed at http://wileyonlinelibrary.com]

A 50‐L funnel‐shaped tank with a bottom draining valve was used to apply chemical treatment (Figure 1). Detached parasites were collected by draining the tank through a cartridge containing a filter paper (130 mm diameter, single‐ply KimNet Wypall X60^®^ cellulose medical wipe, mesh size 40 ± 20 µm). The efficiency of the apparatus for recovering detached parasites was tested in the laboratory prior to the farm trial by repeated flushing and screening of filtrates spiked with a single parasite to determine the probability of detection.

#### 2.3.2 Farm testing

The field test was conducted at a trout restocking farm in the Dorset Stour river catchment in south‐west England. Ten groups of 8–13 fish (mean length 190 mm, mean weight 80 g) from a stock of brown trout infected with a native *Gyrodactylus* spp. were exposed to hydrogen peroxide as detailed in the chemical sampling procedure (Table 2). Groups of 10 control fish from the same stock were netted to a holding container, immediately euthanized by concussion and destruction of the brain and fixed in 100% ethanol for whole‐fish parasite counts in line with the OIE diagnostic manual (OIE, 2018) and the current statutory surveillance procedure conducted by the Cefas Fish Health Inspectorate for *G. salaris* (FHI, 2018). The water temperature remained stable at 8.9°C for the duration of the study.

**Table 2:** Chemical sampling procedure

1	Fill the treatment tank with 10 L of water from the stock rearing tank (i.e. a volume appropriate to accommodate a group of fish for treatment)
2	Add hydrogen peroxide to the tank to make a working solution of 560 ppm
3	Transfer a group of fish directly from the rearing tank and hold in the treatment solution for 3 min (we moved fish with a 5‐mm mesh rubberized net (Team Daiwa, Wishaw, UK) to reduce the likelihood of parasites attaching to the net)
4	*[For assessment of gyrodactylid recovery rate only]* Remove the fish to a holding container, euthanize and fix in 100% ethanol (to establish the number of *undetached* parasites)
5	Drain the treatment solution through the filter cartridge. While emptying, spray a small quantity of water around the inside of the tank to ensure that no parasites are deposited on its surface. If the flow becomes significantly reduced by the accumulation of suspended particulates, close the discharge valve and replace the filter paper
6	Wash through the system with a litre of clean water to flush through any remaining parasites before removing the final filter
7	Store filters in ethanol

Water from the holding containers for both treated and control groups was filtered separately to recover the parasites lost from fish prior to euthanasia and stored separately in ethanol. All filter papers and fish (treated and control) were screened for parasites using a stereomicroscope. Parasites were collected and counted using a pipette and stored in ethanol. The timings for each step of both methods were recorded during the field study and subsequent laboratory processing for comparison.

### 2.4 Statistical methods

All study data were visualized graphically and analysed statistically in R version 3.4.3 (R Development Core Team, 2017). For the efficacy screening study, box plots were used to examine the distribution in the proportion of parasites found in the treatment solutions tested; that is, those removed from hosts (bold lines in box plots are median values, boxes indicate inter‐quartile range, error bars indicate 10 and 90 percentiles, whiskers extend to 1.5 semi‐IQR, and circular symbols indicate outliers). Logistic regression was used to compare the odds of parasites being found off a fish compared with on a fish after exposure to each of the test chemicals. The effectiveness of each chemical at removing parasites from the fish was compared with the proportion of parasites found off fish in the water control (W). This analysis was performed using the ‘glm’ function and assumed a binomial or quasibinomial error distribution (in the case of over‐dispersed data, determined graphically and by comparing the null deviance with the degrees of freedom) and used a ‘log link’ function. Maximal models that included all factors and interaction terms were built in the first instance. Factors or interactions that were not significant (*p* ≤ 0.05) were then removed in a stepwise fashion (least significant first) until only significant terms remained. The reduction in the null and residual deviance observed between the resulting model and the null model was then compared using a chi‐squared test (through the ANOVA function in R) to determine whether the inclusion of the remaining factors led to a significant (*p* ≤ 0.05) improvement in model fit. Diagnostic plots were then used to qualitatively assess model fit. Where outlying points with a high degree of leverage on the model results were identified, the analysis was rerun after their removal, and goodness of fit re‐evaluated. Fish found to be free of parasites after both examination of the body and their treatment solution were also removed from the analysis.

For each trial, the sensitivity of detection was evaluated for each chemical in terms of the probability of detecting at least one parasite from each infected fish by examination of the solution to which they were exposed. Exact confidence intervals around these probability estimates were subsequently computed using the method proposed by Clopper and Pearson (1934).

For the main farm trial, the total number of parasites obtained in each of the ten replicates from the three study groups (pools of control fish, filter papers and the pools of treated fish) were compared graphically and statistically using an analysis of variance (ANOVA) after first transforming the data to normalize the distribution and reduce differences in variability between groups. Graphical comparisons were made using box plots (as described above).

The gyrodactylid recovery rate is the proportion of the total gyrodactylid population on a fish (or group of fish) that is recovered for species identification. The recovery rate of the statutory protocol was estimated by dividing the average number of parasites found on each fish stored in ethanol by the sum of the number of parasites identified in the holding water, the total number of parasites found on filter papers and on the treated fish divided by the total number of fish sampled under the chemical method. The gyrodactylid recovery rate of the non‐lethal (chemical) method was assumed to be represented by the probability of a parasite being removed from fish during the treatment (average number of parasites found on a filter paper divided by the sum of the number of parasites on the filter paper, in the holding water and remaining on the treated fish), multiplied by the probability of recovering a parasite from the system. For each surveillance methodology, 95% confidence intervals around this value of recovery rate were estimated using the methods described by Clopper and Pearson (1934).

Based on the gyrodactylid recovery rates established using the methods described above, estimates were made to assess the sample sizes required to be 95% confident in detecting the parasite in a large population if as few as 0.1% of the population were to harbour 1 or more gyrodactylids. For the statutory method which is based on the screening of individual fish, this was achieved using the Ausvet disease freedom calculator (http://epitools.ausvet.com.au/content.php?page=Freedom), which is based on the methods described by Martin, Shoukri, and Thorburn (1992). For the chemical method, which is based on the detection of the parasite from pools of fish, the Ausvet pooled testing disease freedom calculator (http://epitools.ausvet.com.au/content.php?page=PPFreedom) was used, which is based on the methods described in Cowling, Gardner, and Johnson (1999). Both methods assume 100% test specificity, which in the case of gyrodactylids is a reasonable assumption as identification to the family level is straight forward.

## 3 RESULTS

### 3.1 Chemical review

The literature review identified 86 compounds that have been tested against gyrodactylids or other closely related parasites. These compounds have been evaluated under a total of 406 treatment variations (time, dose, combinations – see Data S1). Twenty‐four compounds were effective against helminth parasites and proved safe to fish under 109 different treatment variations.

### 3.2 Chemical efficacy testing

*Gyrodactylus* spp. are hyperviviparous (Huyse, Audenaert, & Volckaert, 2003) and are known to readily move between hosts (Bakke, Harris, Jansen, & Hansen, 1992). The close proximity between the hosts in the pre‐trial cohabitation phase resulted in a rapid distribution of parasites throughout the wild and laboratory‐reared populations. No significant adverse reactions by the host fish were observed during exposure to any of the chemicals.

#### 3.2.1 Trial 1

**Figure 2:**
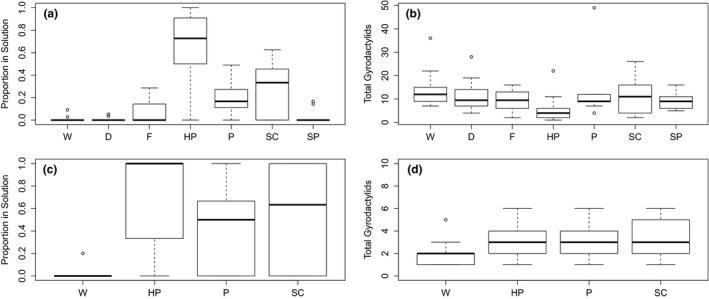
Summary data from Trial 1 (plots a and b) and Trial 2 (plots c and d). Left‐hand plots show the proportion of parasites found in the chemical solution, and right‐hand plots show total *Gyrodactylus* spp. per host (D, dead control; F, formaldehyde; HP, hydrogen peroxide; P, Praziquantel; SC, sodium chloride; SP, sodium percarbonate; W, water control)

With the exception of sodium percarbonate (SP; *p* > 0.3), the chemicals tested all resulted in a significantly higher proportion of parasites being found off the host in solution than observed in the live fish water control group (W) (Figure 2a,b). Hydrogen peroxide was most effective in removing parasites from fish (*p* < 0.001), and the odds of finding a parasite in this treatment solution compared with on a fish were 351 times greater than observed in the water controls (W).

Parasites were significantly more likely to be found in solution when testing large fish compared with small. However, subsequent analysis demonstrated that large fish harboured significantly more parasites than the smaller hosts and that the effect of fish size confounded with the total number of parasites per fish. As the total number of parasites per fish increased, so did the probability of observing parasites in solution. The number of parasites per fish did not, however, interact or confound with the effects of any of the chemical treatments applied. No significant difference (*p* > 0.2) in the proportion of parasites found in solution was observed between the live and dead fish water controls (W and D, respectively). Inclusion of treatment and fish size in the model led to a significant overall improvement in model fit based on the reduction in residual deviance.

#### 3.2.2 Trial 2

Based on the results of Trial 1, the three compounds considered to have the best potential for parasite removal were re‐tested with greater replication (*n* = 20) using larger laboratory‐reared fish (with greater parasite prevalence) to provide a more robust assessment of efficacy (Table 1). A significantly higher proportion of parasites were found off the host in solution in all treatments compared with the water control group (W) (*p* < 0.05, Figure 2c,d). The effects of fish size observed for each treatment were also similar to those observed in Trial 1. Again, hydrogen peroxide was the most effective chemical at removing parasites from the fish (*p* < 0.001), and the odds of finding a parasite in solution compared with on a fish were 160 times greater after treatment than observed in the water controls. Inclusion of treatment in the model led to a significant overall improvement in model fit based on the reduction in residual deviance. Unlike Trial 1, the total number of parasites per fish did not have a significant influence on the likelihood of detecting parasites in solution, possibly due to the lower average number of parasites per fish in Trial 2 compared with Trial 1.

#### 3.2.3 Detection sensitivity

Table 3 shows the number and proportion of parasite‐positive fish per treatment group that were correctly identified as being positive by screening only the solution to which fish were exposed (i.e. at least one parasite was found in solution). Results were relatively consistent (<15% difference) for all the chemicals tested in both trials. None of the chemicals tested provided 100% detection sensitivity, but exposure to hydrogen peroxide was highly effective, and led to between an 89% and 80% detection sensitivity for trials one and two, respectively.

**Table 3:** Parasite detection sensitivity: infected fish that were correctly identified as being positive by screening only the solution to which they were exposed

Chemical	Trial 1	Sensitivity (±95% CI)	Trial 2	Detection sensitivity (±95% CI)
Control 1: Live fish	0/10	0.00 (0.000–0.309)	0/13	0.00 (0.00–0.247)
Control 2: Dead fish	0/10	0.00 (0.000–0.3109)	–	–
Hydrogen peroxide	8/9	0.89 (0.518–0.997)	12/15	0.80 (0.519–0.957)
Formalin	4/10	0.40 (0.122–0.738)	–	–
Praziquantel	7/9	0.78 (0.400–0.972)	9/13	0.69 (0.386–0.909)
Sodium chloride	5/9	0.56 (0.212–0.863)	10/14	0.71 (0.419–0.916)
Sodium percarbonate	2/10	0.20 (0.025–0.556)	–	–

#### 3.2.4 Parasite identification

In all but one case, the morphological characteristics of the hamuli and marginal hooks were consistent with that of *G. gasterostei*. The remaining parasite, although confirmed to be *Gyrodactylus* spp., could not be identified to the species level due to the orientation of the sample's opisthaptor. Eight of the samples submitted for molecular analysis were shown to be a 99% match to *G. gasterostei* isolate Endrick Accession number EF446728. One sample could not be identified and was assumed not to be of the genus *Gyrodactylus* (sequencing analysis produced no result, and no further work could be done on this isolate).

### 3.3 Field validation

#### 3.3.1 System validation

The average time required to drain 50 L of clean water from the treatment tank through the filter mechanism was 15 min. This could be achieved using a single 30‐µm filter mesh. The observed probability of recovering a single (dead, fixed) parasite spiked into the system was *p* = 1.00 (the parasite was recovered from all replicates, lower and upper 95% CIs = 0.69 and 1.00, respectively) based on the 10‐replicate test.

#### 3.3.2 Farm testing

The fish showed no adverse reaction to the treatment and did not appear to be stressed by the procedure. Under field conditions, the addition of fish resulted in the treatment solution becoming turbid and the presence of suspended particles increased the time required to filter the first replicate to 60 min. Subsequent replicates were therefore filtered using a coarser grade mesh (40 ± 20 µm). Although this significantly improved filtration rate, it was still necessary to replace filters to complete the procedure effectively (Table 4).

**Table 4:** Practical summary of farm trial exposure replicates and parasites recovered by chemical exposure and filtration (filters and treated fish) and whole‐body examination (control fish)

Replicate	Groups exposed to hydrogen peroxide				Controls
	Filters required	Time to drain (min)	Parasites recovered (and numbers of fish per replicate)		
			Filters	Fish	Fish
1	5^a^	60	169	14 (13)	181 (10)
2	3	26	179	9 (10)	62 (10)
3	2	11	303	15 (10)	165 (10)
4	2	8	207	9 (8)	169 (10)
5	2	9	249	8 (11)	77 (10)
6	2	8	252	10 (9)	173 (10)
7	2	7	198	8 (8)	170 (10)
8	3	7	273	4 (8)	167 (10)
9	2	9	239	17 (10)	89 (10)
10	3	8	185	140 (11)	125 (10)
	Mean	225	24	138	
	*SD*	44	41	46	

a Replicate 1 with 30‐µm filters, subsequently 40‐µm filters were used to reduce drain time.

**Figure 3:**
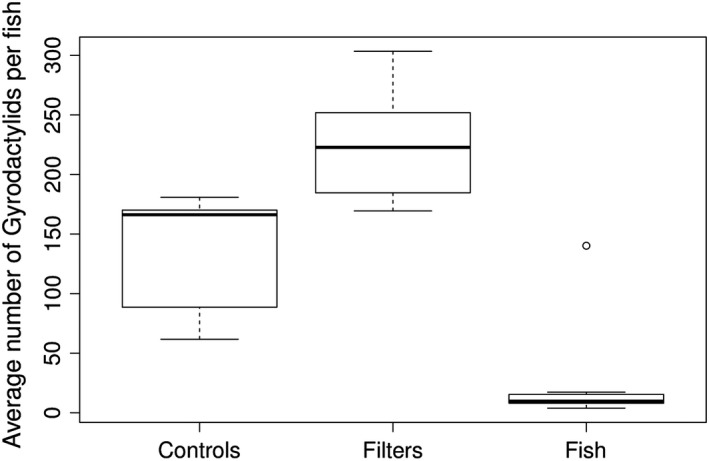
The number of gyrodactylid parasites recovered from fish across replicate samples by filtration following hydrogen peroxide treatment (Filters), compared with those remaining on fish (Fish) and numbers detected by the whole‐fish sampling methodology (Controls)

Significantly more parasites were recovered after exposing fish to hydrogen peroxide than by direct whole‐body examination of killed fish (mean: 225 vs. 138 parasites per fish, respectively, *p* < 0.05, Table 4 and Figure 3). Subsequent whole‐body examination of treated fish confirmed that an average of 24 parasites remained attached to the fish after 3‐min exposure to hydrogen peroxide, which was significantly fewer than found on the fish screened by whole‐body examination (mean: 24 vs. 138 parasites per fish, respectively, *p* < 0.05, Figure 3). When comparing the filtrates from the water used to hold control and treated fish prior to culling, a total of 6,030 (6,030/100 = 60.3 per fish) and 1,671 (1,671/98 = 17.1 per fish) parasites were found, respectively.

Using the total number of parasites obtained by chemical exposure (225 per fish on the filters, 24 remaining on each fish, 17 in the holding water per fish = total 266 per fish), a relative gyrodactylid recovery rate can be estimated for both methods. For chemical exposure, the recovery rate is 225/266 = 84.6% (95% CI: 79.7%–88.7%), achieved by screening filters alone. This needs to be multiplied by the probability of recovery from the exposure apparatus, which based on the preliminary efficacy trials was one, thus leaving the overall recovery rate unchanged. The control fish were sampled from the same tank population, so if we assume that the best estimate of total parasite number per fish was achieved using the chemical method, the same numerator can be used to derive the gyrodactylid recovery rate for screening the fish alone by whole‐body examination: 138/266 = 51.9% (95% CI: 45.7%–58.0%).

The gyrodactylid recovery rates allow the sample sizes required to detect parasite presence in a large fish population to be determined with an associated confidence. Assuming a 51.9% gyrodactylid recovery rate for whole‐body examination, it is estimated that 114 fish would need to be sampled to be 95% confident of detecting the parasite if 5% of the population harboured at least one gyrodactylid (576 fish would need to be sampled if 1% were infected with at least 1 parasite, Table 5). For chemical exposure, assuming a gyrodactylid recovery rate of 84.6% and a pool size of 10 fish per treatment, 8 pools of 10 fish (80 fish total) would achieve the same level of confidence (Table 6). If the pools of fish per treatment are increased to 30, three batches of fish (90 fish total) would need to be sampled.

**Table 5:** Sample sizes required for whole‐body examination to be 95% confident of detecting a parasite if one or more are present at different prevalence across a large (infinite) population of fish (assumes sensitivity = 51.9% and specificity = 100%)

Prevalence	Sample size
0.001	5,771
0.005	1,153
0.01	576
0.02	288
0.03	191
0.04	143
0.05	114
0.1	57
0.2	28

**Table 6:** Replicate samples required (i.e. number of pools, assuming different pool sizes for the chemical method) to be 95% confident of detecting the parasite if one or more are present at different prevalence across a large population of fish (assumes sensitivity = 84.6% and specificity = 100%)

Pool size	Prevalence							
	0.001	0.005	0.01	0.02	0.03	0.04	0.05	0.1
1	3,540	707	353	176	117	88	70	34
2	1,770	354	177	88	59	44	35	18
3	1,181	236	118	59	40	30	24	12
4	886	177	89	45	30	22	18	9
5	709	142	71	36	24	18	15	8
10	355	71	36	18	12	9	8	4
15	237	48	24	12	9	7	5	3
20	178	36	18	10	7	5	4	3
25	142	29	15	8	5	4	4	2
30	119	24	13	7	5	4	3	2
40	89	18	10	5	4	3	3	2
50	72	15	8	4	3	3	2	2
100	36	8	4	3	2	2	2	2

#### 3.3.3 Resource comparison of surveillance methods

**Figure 4:**
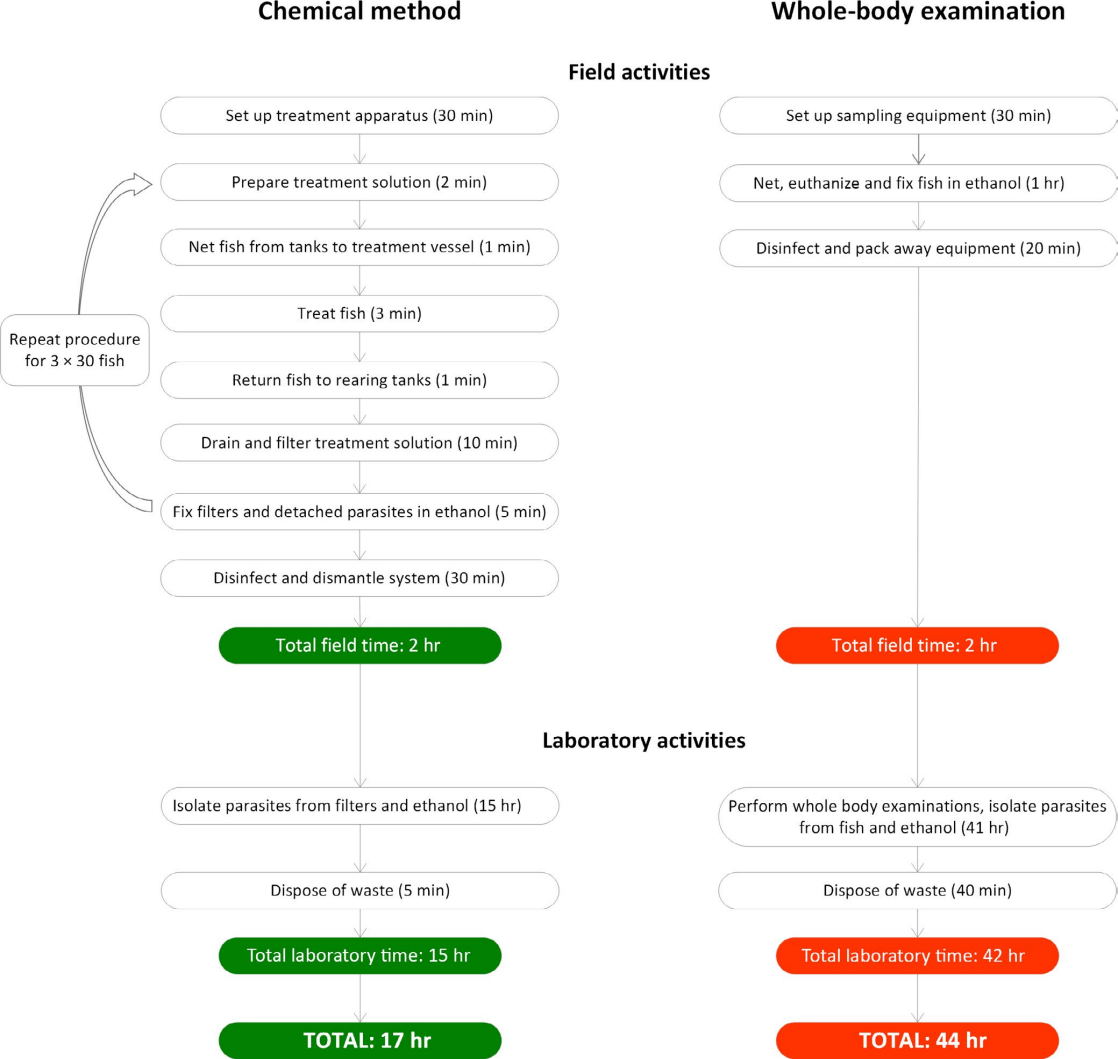
A comparison of the effort required to collect material to determine the infection status of a farmed fish population assuming a design prevalence of 1% with 95% confidence using chemical exposure and whole‐body examination (current statutory method). Assumptions: (a) Chemical procedure 90 fish required for 95% confidence (3 batches of 30 fish), 100 parasites per fish, 6 s to collect each parasite from filtrate; (b) Statutory procedure: 114 fish required for 95% confidence, 100 parasites per fish, 13 s to collect each parasite [Colour figure can be viewed at http://wileyonlinelibrary.com]

Approximate timings for each of the main steps of both procedures were recorded during the study, which were used to estimate the effort required to collect material to determine the infection status of a farmed fish population, assuming a design prevalence of 1% with 95% confidence (Figure 4). The time required for the field activities of both methods was similar (around 2 hr). However, considerable time is subsequently saved in the laboratory as screening (114) fish is replaced by examining less than 10 filters and approximately 300 ml of ethanol containing suspended parasites. It is estimated that the total time to isolate parasites for this surveillance task would be 17 and 44 hr using the chemical exposure and whole‐body examination procedures, respectively.

## 4 DISCUSSION

In the event of an incursion of *G. salaris* into the UK, the testing of trout farms will be an important component of a wider catchment‐level programme of surveillance required to establish the distribution of the parasite. The infection status of individual farm sites is important as trade from farms where *G. salaris* may have been introduced would be restricted until freedom has been demonstrated. The spread of a *G. salaris* parasite among different farmed and wild salmonid species and the magnitude of its impact on wild Atlantic salmon will be determined by the strain that is introduced. A variety of strains of *G. salaris* are known to be present throughout its current geographic range (Hansen et al., 2003, 2007; Meinilä et al., 2004). Although the majority of these are highly virulent against Atlantic salmon, some strains that exhibit limited reproduction and are non‐pathogenic to Atlantic salmon have been isolated from other host species, including rainbow trout (Lindenstrøm et al., 2003) and Arctic charr (Olstad et al., 2007), which are more susceptible to infection. Establishing the infection status and, more specifically, demonstrating freedom from a strain of *G. salaris* pathogenic to Atlantic salmon in rainbow and brown trout, with low levels of prevalence and abundance, presents serious challenges (Peeler & Oidtmann, 2008). This study aimed to identify a compound that could be used in a non‐lethal procedure to remove gyrodactylid parasites from fish hosts and develop a robust method to rapidly recover detached parasites that can be used to screen the large numbers of fish that would be required for testing non‐primary hosts.

Over 80 compounds were reviewed that have been tested against gyrodactylids or closely related parasite species (see Data S1). Of these, five safe and relatively fast‐acting compounds were identified which are already available for use in fish systems and were considered to offer the greatest potential for further study: formalin, hydrogen peroxide, Praziquantel, sodium chloride and sodium percarbonate. Hydrogen peroxide proved to be the most effective option. Aquarium trials demonstrated that short‐duration chemical exposure is effective in removing gyrodactylid parasites from stickleback hosts, even when present in low numbers, with a detection sensitivity of 80%–89% for fish harbouring an average of three parasites.

Other compounds were considered, including aqueous solutions of aluminium which have been demonstrated in several laboratory studies to kill gyrodactylids (reviewed by Schelkle, Shinn, Peeler, & Cable, 2009) and are currently being used in infected Norwegian rivers in combination with acidification (using sulphuric acid) to control the parasite (Hytterød et al., 2018). Although safe for salmonids, it is not fast enough acting for our requirements (parasites were eliminated from salmon after 4 days in trials; Soleng, Poléo, Alstad, & Bakke, 1999). A number of licensed veterinary medicines proven to be effective and fast acting against gyrodactylids could possibly be used under the veterinary cascade (Anon, 2013), including, *inter alia*, Closantel, Fenbendazole, Parbendazole and Triclabendazole, compounds that have been used to treat a wide range of flukes, roundworms and myiases (e.g. toxocara and fascioliasis) in domestic and farmed animals (Data S1). However, their use would result in a 500‐degree‐day withdrawal period being placed on treated fish. This may be acceptable for fingerlings, but not for fish approaching market size, as their consumption would be prohibited until this time had elapsed (VMD, 2015). Furthermore, the use of chemicals with medical or veterinary application should be avoided, if possible, to mitigate the potential for the development of pest‐species resistance if the compound were to be released into the aquatic environment.

The farm study demonstrated that endemic gyrodactylid parasites can be recovered from commercially reared salmonids, following treatment with hydrogen peroxide, under field conditions. The chemical method achieved a 33% improvement in gyrodactylid recovery rate (84.6%) compared with that of whole‐body examination alone (51.9%), and the procedure historically conducted by the Cefas Fish Health Inspectorate for surveillance in England and Wales. The total parasite count estimated by the chemical method was 25% higher than that of whole‐body examination, and this may in part be explained by parasites that were attached within the buccal and gill cavities of fish (Grano‐Maldonado, 2014). These may be hidden to screeners conducting physical examinations but are detached and recovered following exposure to hydrogen peroxide (this may be influenced by the experience of the screener(s)). Our approximations of gyrodactylid recovery rate for both methods were therefore based on the best estimate of mean total parasites per fish using the chemical method. However, it is likely that this is still an underestimate, as some parasites may also have remained attached within the buccal and gill cavities of chemically treated fish and would therefore have not been accounted for by this method either. This may be less of an issue for *G. salaris*: as it has a preference for attachment on the dorsal and pectoral fins, occurrence in the buccal and gill cavities is not common (Bakke et al., 2007; Bakke, Harris, & Cable, 2002).

The high numbers of parasites recovered from containment waters indicate that a significant proportion may be lost from surveillance efforts through the handling of fish. Handling fish is unavoidable in any sampling procedure, but is much reduced prior to parasite collection using the chemical method. The loss of parasites through handing prior to euthanasia has important implications for historic assumptions made about the efficiency of parasite recovery and potential impact on surveillance sensitivity (particularly at low levels of prevalence), which to our knowledge has not been rigorously tested.

The recovery rate of the chemical method on‐farm was consistent with the results of the aquaria trials (stickleback model), that is 84.6% versus 80%–89%, respectively. The close similarities in biology of the *Gyrodactylus* genus suggest it is highly unlikely that *G. salaris* would respond differently to the procedure. However, the sensitivity of *G. salaris* to hydrogen peroxide should be experimentally confirmed. It would be undesirable to import live parasites for this purpose into the UK where it is not currently present, so the possibility of further testing in an infected territory (e.g. Norway or Denmark) should be explored.

Peeler and Oidtmann (2008) detail a 3‐stage approach to structuring a survey on a rainbow trout farm which determines required sample sizes for rearing units (tanks or cages), fish within units and then parasites on individual fish to demonstrate freedom from *G. salaris* which may be present in mixed infections with other, non‐pathogenic, gyrodactylid species. When using the chemical approach, we simplify the survey structure by effectively sampling the tank‐level parasite population directly (assuming fish are selected at random for treatment). We address only the determination of fish numbers required to ensure that pathogenic gyrodactylids are collected by the procedure, if present in a tank population. These estimates were informed by observed gyrodactylid recovery rates (not previously considered) and a conservative estimate of expected prevalence of *G. salaris* at the fish level (5% – based on observed prevalences in Norway; Bakke et al., 2002; Bakke et al., 2007), which was assumed to be independent of the presence of other gyrodactylid species. However, as seen in the farm trail conducted for this study, the subsequent selection of parasites for screening may be greatly complicated by the presence of large numbers of gyrodactylids of endemic species. Further work is now required to review the overall approach to farm surveys for *G. salaris* incorporating the chemical procedure for collection. Specifically, attention should be directed towards the selection of recovered parasites for diagnostic screening, the revaluation of test sensitivity to account for the efficiency of parasite collection (diagnostic sensitivity) and efforts to locate and review (possibly unpublished) data on abundance and prevalence of mixed gyrodactylid infections on trout farms in *G. salaris* infected territories which may help to improve estimation of test design prevalences.

A comparison of timings for the two methods indicates that there is scope for significant savings in adopting the chemical screening method, with the isolation of parasites from samples returned to the laboratory being completed in approximately a third of the time compared with that of whole‐body screening (although the latter will be largely determined by parasite abundance and the experience of screeners). Granted the task here was to recover all the parasites to estimate the recovery rate, and comparable efficiencies will be realized when sampling parasites from pooled populations for diagnostic testing. There are further health and safety and cost benefits associated with the quantity of ethanol used for sample storage: in this study, approximately 80 L of ethanol was used to store 100 fish for the control method, whereas approximately 1 L was used to store 26 filters.

Although the driver for this work was the necessity for an ethical and workable technique for trout farms, the methodology developed can also be applied to surveillance in wild salmon populations. Cefas works with the Environment Agency's juvenile salmon population monitoring programme, to conduct a rolling programme of surveillance for *G. salaris* in England and Wales, sampling 10 river catchments per year. Historically, riparian owners have been reluctant to allow even small numbers of salmon parr to be killed for this purpose, particularly from commercially valuable or threatened salmon stocks. As a result, the sensitivity of the programme, at a national level, is low. The introduction of a non‐destructive approach to testing, however, potentially permits more fish to be screened and more sensitive populations to be included in the routine peacetime surveillance, ultimately increasing confidence in freedom from *G. salaris*. Non‐destructive parasite collection also opens the door to a wider investigation of general gyrodactylid ecology, allowing the characterization of ‘normal’ gyrodactylid occurrence in healthy salmon, and cohabiting species, in different river systems. This may provide valuable background data to inform the investigation of introduced species, the emergence of new strains or in modelling their impact.

The key objective in an outbreak situation would be to protect the *G. salaris*‐free status of important salmon rivers, making wild salmon the primary focus of emergency surveillance. The consequences of incorrectly concluding that a river is free from infection are potentially serious and irreversible; however, surveys can only substantiate that a parasite is not present above a specified minimum detectable prevalence (the design prevalence, see Cameron & Baldock, 1998a, 1998b). The method described here will enable rapid screening of multiple sampling locations across river catchments and provides the best way forward in the design of sampling strategies needed to demonstrate freedom with a high level of confidence (e.g. 99%). We recommend that the method is refined and fully utilized through incorporation into routine surveillance to develop competencies in peacetime in readiness for emergency response.

The application of environmental DNA (eDNA) analysis in monitoring programmes offers potential benefits including the detection of rare, cryptic or otherwise hard to survey aquatic organisms, multisite screening with reduced effort and fewer sampling biases compared with the collection of specimens (Bass et al., 2015). Reliable tools are on the horizon, and Rusch et al. (2018) have already developed a protocol for the detection of *G. salaris*. However, the method presented in the current study does still offer significant advantages over an eDNA approach for the detection of gyrodactylids. Firstly, it is likely to be more sensitive – the sensitivity of eDNA analysis (and the likelihood of false‐negative results) would be difficult to quantify, thus reducing the confidence in establishing freedom from infection. In addition, as all parasites are recovered, they can be identified to the species‐ or even to strain‐level if needed, this would include previously unknown species or strains which could not be detected by specific eDNA assays. Furthermore, evidence of infection with notifiable pathogens provided by eDNA methods must (at present) be confirmed by diagnostic tests prescribed by current legislation and thereby require actual parasites. eDNA methods do, however, have great value in directing the search for parasitic pathogens and provide an important complement to conventional survey work.

## 5 CONCLUSIONS

This study demonstrated that hydrogen peroxide bath treatment may be successfully applied to the surveillance of gyrodactylid parasites and established as a non‐lethal method for sampling farmed and wild fish stocks. Hydrogen peroxide is already licensed for the treatment of fish for ecto‐parasites and is used routinely by fish farms and by the aquarium trade with no impact on fish. It is also safe for operators (subject to appropriate risk assessment) and the environment (i.e. it does not break down into toxic residues). This approach has the potential to reduce resources required to collect and isolate parasites for diagnostic testing and improve the sensitivity and confidence of surveillance programmes designed to demonstrate freedom from disease, thus underpinning a robust and defensible surveillance strategy for *G. salaris* for the UK aquatic animal disease contingency plan.

## ENDNOTES

1 STARFISH is a resource jointly owned by Cefas, Environment Agency, Welsh Government and Defra, to manage all data relating to Aquaculture Production Business authorisation and registration, aquatic animal imports and exports, the rearing and holding of non‐native species, and statutory aquatic disease testing and control (System for Tracking And Recording FIsh and SHellfish).

2 The Cefas Fish Health Inspectorate acting, on behalf of Defra and the Welsh Government, has statutory responsibility for the control of notifiable and emerging disease in aquatic animals in England and Wales.
